# Editorial: Implications of metal uptake and resistance in plants for phytoremediation, biofortification and food safety concerns, volume II

**DOI:** 10.3389/fpls.2023.1334016

**Published:** 2023-11-22

**Authors:** David W.M. Leung

**Affiliations:** School of Biological Sciences, University of Canterbury, Christchurch, New Zealand

**Keywords:** arbuscular mycorrhizal fungus (AMF), cadmium (Cd), climate change, fly ash, global warming, heavy metal ATPase (HMA), quantitative trait loci (QTL), selenium (Se)

Phytoremediation has been viewed as a way of using metal- (loid-) resistant plants to remove toxic metals (loids) from the environment to help to solve the following problems. Plants have the intrinsic properties to absorb elements essential for plant growth from the environment, but toxic metals (loids) can also be taken up by plants. The toxic metals (loids) are largely devoid of known physiological functions in plants but could adversely affect crop plant productivity and therefore pose threats to food security. The absorption and translocation of toxic metals (loids) into the edible parts of crop plants could also pose food safety and public health concerns. The contributions in this Special Topic “Implications of metal uptake and resistance in plants for phytoremediation, biofortification and food safety concerns, Volume II” included a detailed examination of genes involved in metal uptake, translocation, and resistance in plants in relation to phytoremediation and development of metal-safe cultivars. In addition, there were new findings from research on plants in treatment with metals (loids) in combination with additional environmental challenges/ecosystem interactions. Furthermore, novel research dimensions were also highlighted in this Special Topic including biofortification of micronutrients by plants not only for the purpose of helping to solve the problem of micronutrient deficiency in human nutrition, but also for counteracting the potential problem of absorption of toxic metals by plants, and new large-scale use of fly ash, a metal-rich substrate.

Bayanati et al. first examined two foci of research separately: (a) the importance and methods of biofortification of micronutrients such as zinc (Zn) and selenium (Se), and (b) the uptake and the undesirable effects of toxic heavy metals such as cadmium (Cd) and mercury (Hg) on food plants and therefore human health. Different strategies to reduce accumulation of Cd, lead (Pb), nickel (Ni) etc. in plants were then discussed. These include taking advantage of the interactions between Se and Cd by biofortifying crops in Se-enriched soil, and using Zn biofortification by plant transformation to reduce Cd uptake and accumulation in plants. In addition, medicinal plants and metallothionein genes were highlighted as useful genetic resources for biofortification and phytoremediation.

Ai et al. examined Cd uptake into rice plants and a long list of gene families, including the heavy metal ATPase (HMA) gene family and the natural resistance-associated macrophage proteins (NRAMP) gene family, involved in Cd detoxification and translocation within rice plants. In addition, the genomic variations in Cd uptake, translocation, detoxification and tolerance in different rice plant varieties from quantitative trait loci (QTL) analysis were evaluated. There was also discussion about the knowledge gaps in relation to the development of useful low Cd-accumulating rice varieties with high yields and good grain qualities for human consumption as well as Cd-tolerant rice varieties to help to mitigate Cd pollution.

Li et al. evaluated the protection of foliar application (biofortification) of silicon (Si) against stem rust disease development in oat leaves. The findings give support to the hypothesis about the protective effect of Si on photosynthesis performance as an underlying protective mechanism against stem rust disease development in oat. In particular, new information was obtained about the reduction, by Si application, in the impairment of the different parameters associated with the performance of photosynthesis such as stomatal conductance, maximum rate of photosynthesis, chlorophyll a fluorescence parameters, and the contents of photosynthetic pigments in oat leaves inoculated with stem rust.

Gao et al. conducted investigations into the protective effects of *Glomus mosseae* (an arbuscular mycorrhizal fungus, AMF) on alfalfa against Cd exposure under elevated air temperature condition (Cd + T). This was rarely investigated as prior studies mostly examined separately the effects of AMF on plants exposed to either heavy metal or high temperature stress condition, but not under the combined conditions. Many interesting findings were reported including higher root biomass and Cd content in the roots under Cd + T when the plants were inoculated with AMF compared to without AMF. In particular, this study is of relevance to the need for a better understanding of the effect of AMF to help phytoremediation of heavy metal pollution under global warming scenarios.

Liu et al. evaluated the possibility of utilising raw fly ash produced in large quantity from coal burning as the bulk of soil matrix in remediation and large-scale environmental restoration of spent mine sites. Raw fly ash was found to contain various heavy metals such as Ni, Zn, Pb, Chromium (Cr), Cd, etc. However, with reference to the risk assessment indices of heavy metals for ecological restoration by the Ministry of Civil Affairs of the People’s Republic of China, 2021, the heavy metal content of the fly ash in this study was considered to be feasible for the purpose of ecological restoration of mine sites. In this study, the effects of different factors in various combinations on the characteristics of the fly ash as a functional soil substrate and different parameters of oat plant growth were investigated. The findings are useful to the further development of fly ash, an environmental waste, into the main functional soil substrate that can support plant growth.

Research into the molecular mechanisms underpinning metal (loid) uptake, translocation and resistance in plants is central to a better fundamental understanding of phytoremediation, biofortification and development of metal-safe crop cultivars. The contributions in this Special Topic showed some new research dimensions to increase the breath and depth of our understanding of metal biology in plants when the complex interactions in the ecosystem and climate change had also been taken into consideration.

## Author contributions

DL: Conceptualization, Writing – original draft, Writing – review & editing.

